# Very Early Onset of Fistulizing Inflammatory Bowel Disease With RIPK1 Mutation: A Case Report

**DOI:** 10.7759/cureus.55708

**Published:** 2024-03-07

**Authors:** Rola K Bsharat, Mahmoud E AbuBshara, Islam H Karajeh, Amal F Bast, Taima M Aljabari, Osama Q Qumsieh, Haytham M Abumohsen

**Affiliations:** 1 Health Sciences, Palestine Polytechnic University, West Bank, PSE; 2 Health Sciences, An-Najah National University, Nablus, PSE; 3 Pediatric Surgery, Al Ahli Hospital, Hebron, PSE; 4 Emergency Medicine, Palestinian Ministry of Health, Jenin Governmental Hospital, Nablus, PSE; 5 Medicine and Surgery, Palestinian Ministry of Health, Tubas Governmental Hospital, Nablus, PSE

**Keywords:** perianal disease, pediatric inflammatory bowel disease, crohn’s disease, monogenic, ripk1, veo-ibd, ibd

## Abstract

Infantile inflammatory bowel disease (IBD) is a very rare subgroup of IBD that develops in children younger than two years with genetic susceptibility, especially in those with monogenic defects. This type, when compared with IBD in older children, is more resistant to conventional medical treatment and presents with more complications that require more surgical interventions.

Our patient is a male with first-degree consanguineous parents. He was 16 months old when he presented with multiple perianal fistulas, fissures, abscesses, diarrhea, fever, and failure to thrive. He underwent a protective double-barrel ileostomy and surgical repair of the perianal disease.

Crohn’s disease was confirmed after endoscopy and biopsy. A genetic workup was done and revealed receptor-interacting protein kinase 1 (*RIPK1*) mutations.

Conventional pediatric IBD treatment was initiated after surgery, including tumor necrosis factor antagonist adalimumab 40 mg subcutaneously weekly for five months. Despite treatment, he presented with dysuria and a colovesical fistula. The patient underwent secondary surgical repair.

## Introduction

Crohn’s disease (CD), a type of inflammatory bowel disease (IBD), is a chronic disorder with an unknown mechanism. It is a destructive disease that affects any part of the gastrointestinal tract, causes inflammation of all the layers of the affected bowel, and tends to cause fistulas [1].

Although CD mainly affects adults, 25% of patients develop symptoms during childhood or adolescence. Its incidence has been increasing over the last few years [2]. However, very few cases have reported an infant with CD, and epidemiological studies from North America and Europe report that fewer than 1% of children with IBD present before one year of age [3].

IBD that develops before two years of age is referred to as infantile IBD, and if it occurs before six years of age, it is known as very early-onset inflammatory bowel disease (VEO-IBD) [4]. It has a strong genetic component, and approximately 50 mutations have been identified using advanced genetic sequencing techniques [5]. Most patients respond well to conservative management such as adalimumab, but surgical intervention is required in case of intestinal obstruction, perforations, and abscesses with fistula formation [6].

Here, we report a sporadic case of a 16-month-old child with severe fistulizing CD secondary to receptor-interacting protein kinases 1 (*RIPK1*) mutation showing poor response to conservative management. The patient underwent surgical interventions including fistulotomy, fistulectomy, and ileostomy [7].

## Case presentation

A 16-month-old male patient with an uneventful perianal disease history presented to the hospital with perianal openings and stool discharge from those openings for about two weeks. Regarding perinatal history, the child had been born to a 28-year-old mother who was primigravida and was delivered by normal vaginal delivery without any post-natal complications or need for neonatal intensive care unit admission. The openings appeared acute and progressive in course regarding the number and size in association with weight loss and failure to thrive. The patient had a history of chronic diarrhea since the age of nine months, for which the family sought medical attention with no definitive diagnosis. During this period, the family was reassured that it could be viral gastroenteritis or that the child’s gut was still developing at this age and would improve over time. Moreover, stool analysis was done multiple times but the reports found no bugs.

**Figure 1:**
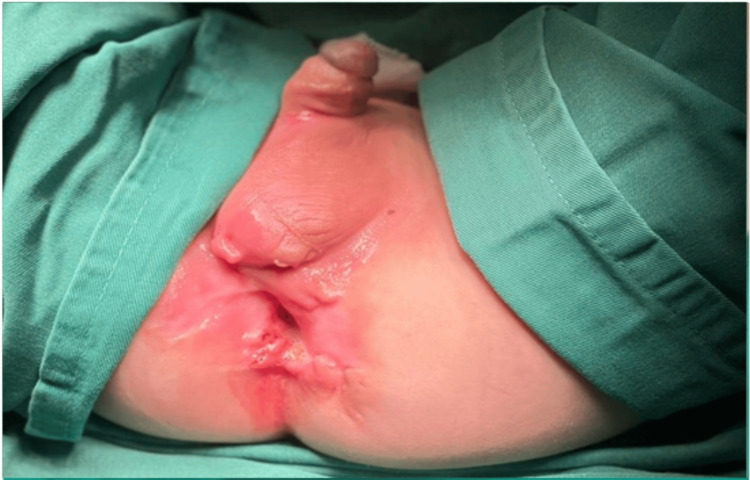
Multiple perianal fistulas.

On physical examination, the patient looked pale and cachectic. There were multiple perianal openings with varying sizes and deep penetration surrounded by erythema with tenderness on palpation (Figure 1). His labs on admission including complete blood count are shown in Table 1. C-reactive protein (CRP) was 255 mg/L. Regarding the vasculitis profile, cytoplasmic antineutrophil cytoplasmic autoantibody was negative at <3 U/mL, perinuclear antineutrophil cytoplasmic antibody was negative at <3 U/mL, and glioblastoma multiforme was negative at <3 U/mL. The indirect Coombs test was negative, and clinical chemistry (blood urea nitrogen (BUN) = 3 mg/dL, creatinine = 0.34 mg/dL, serum glutamic-pyruvic transaminase = 40 U/L, serum glutamic-oxaloacetic transaminase = 74 U/L) was within the normal range. Albumin was 2.7 g/dL (normal range = 3.5-5-5 g/dL). Serum electrolytes showed the following findings: Na = 136 mEq/L, Cl = 101 mEq/L, and K 3.3 mEq/L.

**Table 1:** Laboratory workup of the patient at his initial admission. WBC = white blood cell; RBC = red blood cell; MCV = mean corpuscular volume; MCH = mean corpuscular hemoglobin; MCHC = mean corpuscular hemoglobin concentration; RDW-CV = red cell distribution width - coefficient of variation; MPV = mean platelet volume

Component	Unit	Patient’s result	Standard range
WBC count	10^3^/µL	26	4.5–11
RBC count	10^3^/µL	4.07	3.5–5.5
Hemoglobin	g/dL	8.8	12–15
Hematocrit	%	34.4	36–48
MCV	fL	75	79–101
MCH	pg	28.3	25–35
MCHC	g/dL	33.4	31–37
RDW-CV	%	13.9	11–16
Platelet count	10^3^/µL	266	150–420
MPV	fL	10.6	7–10
Neutrophils	%	60.2	40–74
Lymphocytes	%	29.1	14–46
Monocytes	%	10.68	4–13

**Figure 2:**
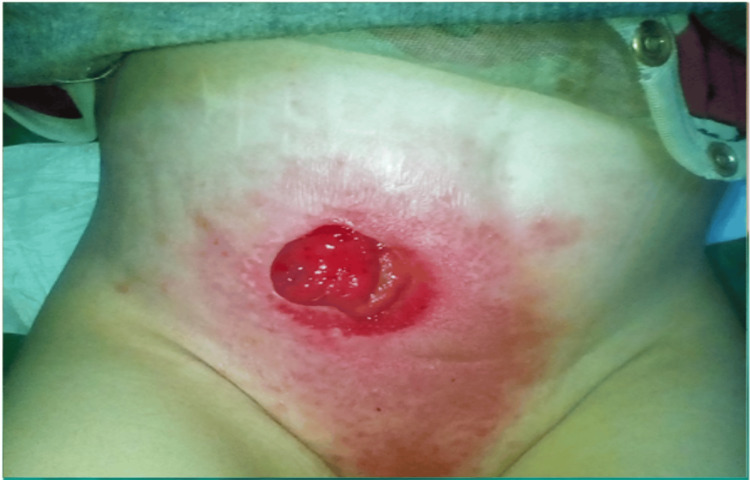
Ileostomy.

**Figure 3:**
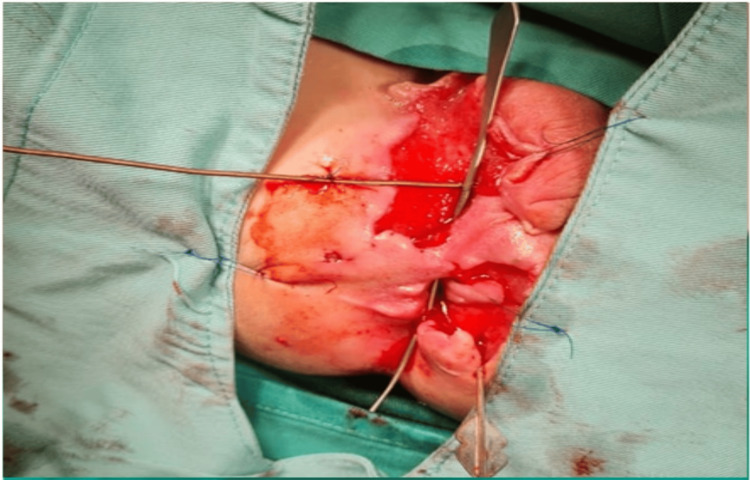
Double-barrel fistulotomy.

The patient was suspected to have infantile CD. A colonoscopy was done on April 24, 2021, and showed severe disease at the ileocecal valve, ascending colon, and sigmoid colon, and pseudopolyps in the ilium with a fistula at the distal sigma (Table 2). The patient underwent a double-barrel ileostomy which showed pancolitis with a thickened wall of the entire colon and involvement of 10 cm in the terminal ileum (Figures 2, 3).

**Table 2:** Colonoscopy findings according to the radiology report. Impression: Severe disease at the ileocecal valve, ascending colon, and sigmoid colon, with fistulas at the distal sigma.

Site in the bowel	Findings
Anal canal	Normal
Rectum	Normal
Sigmoid colon	Severe disease with deep ulcerations and fist
Descending colon	Normal
Splenic flexure	Normal
Transverse colon	Normal
Hepatic flexure	Normal
Ascending colon	Deep ulcerations with edema and erythema
Ileocecal valve	Deep ulcerations with edema and erythema
Cecum	Normal
Ileum	Normal

Follow-up labs after surgery showed a hemoglobin of 10.3 g/dL, white blood cell count of 14,000/µL (neutrophils = 53.2%, lymphocytes = 39.6%, and eosinophils = 2.1%), platelets of 622,000/µL, and CRP of 50 mg/L. Regarding serum electrolytes, Na was 147 mEq/L, K was 3 mEq/L, and Cl was 122 mEq/L. BUN was 3 mg/dL, and creatinine was 0.32 mg/dL. The patient was discharged on Pentasa (mesalamine) 25 mg (1×2), Humera (adalimumab) 40 mg 0.4 cc subcutaneously weekly, and Imuran (azathioprine) 50 mg (¼×1).

After five months, the patient presented with anal and perianal discharge with a minimal amount of pus. Upon examination, the patient appeared well, with an active soft lax abdomen. The double-barrel ileostomy was functioning properly. Perianal examination showed multiple ulcers and openings in the perianal area. Labs on admission were a hemoglobin of 13.5 g/dL, white blood cell count of 9,000/µL (neutrophil = 39.1%, lymphocytes = 47.5%, and eosinophils = 4.5%), and platelets of 328,000/µL. Regarding serum electrolytes, Na was 137 mEq/L, K was 4.2 mEq/L, and Cl was 105 mEq/L.

His doctor decided for him to undergo an examination under general anesthesia with fistulotomy and fistulectomy. During the examination, multiple perianal fistulas were noted at 11, 1, 3, and 5 o'clock positions with multiple skin tags and severe anal stenosis.

The patient was on maximum treatment for CD since diagnosis (Pentasa, Imuran, and Humera), without improvement. Subsequently, the patient was readmitted after seven months, complaining of multiple openings with pus discharge and erythema, despite prior surgical intervention and medical treatment. Labs at this time were a hemoglobin of 11.06 g/dL, white blood cell count of 11,000/µL (neutrophil = 69.6%, lymphocytes = 20.8%, and eosinophils = 0.49%), and platelets of 342,000/µL. BUN was 5 mg/dL, and creatinine was 0.47 mg/dL. Regarding serum electrolytes, Na was 139 mEq/L, K was 4.5 mEq/L, and Cl was 109 mEq/L.

**Figure 4:**
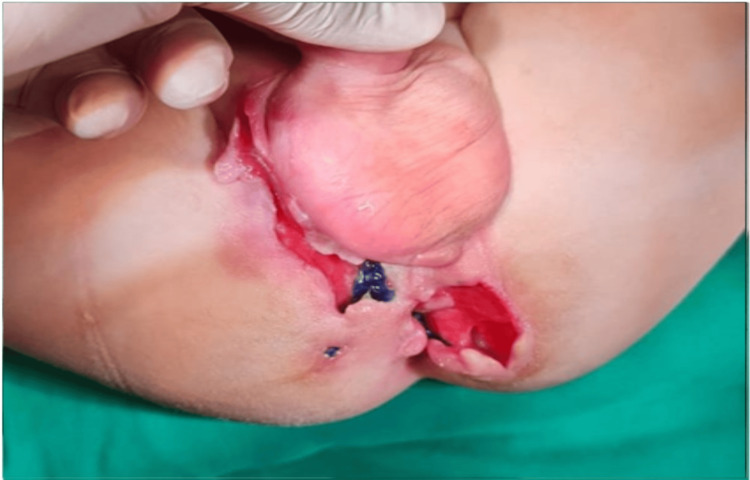
Fistula repair with flap.

Another surgery was done due to a rectourtheral fistula. The diagnosis was confirmed by injecting methylene blue, which came out from the anal sphincter. Other fistulas were treated by rectal mucosal advancement flap to cover the opening of the fistula after the insertion of a mesh inside the fistula (Figure 4). The colonoscopy showed severe disease at the ileocecal valve, ascending colon, and sigmoid colon, as well as pseudopolyps in the ilium with a fistula at the distal sigma. One year later, a follow-up colonoscopy revealed a distorted anus with normal colonic mucosa. The patient is now doing well, has had no recent attacks, and continues to follow up with his pediatric gastroenterologist regarding his medications and his pediatric surgeon regarding his distorted anus and perianal fistulas.

## Discussion

Monogenic mutations have been linked to IBD. One of these mutations is *RIPK1 *mutation which should be suspected in patients with VEO-IBD presenting with severe forms of inflammation and fistulas.

RIPK1 is a part of the receptor-interacting protein (RIP) family of serine/threonine protein kinase. *RIPK1*-encoded protein acts as an important regulator of programmed cell death (apoptosis), inflammation, and necrosis via activation of surface receptors, such as tumor necrosis factor (TNF) receptor 1, toll-like receptors (TLR3, TLR4), and interferon receptors. On the other hand, the loss of function of this gene is associated with immunodeficiency, multisystemic diseases with inflammation such as early-onset IBD, arthritis, and intestinal epithelial cell dysfunction [7,8].

To date, 16 patients with *RIPK1 *mutation have been reported in the literature. Most developed colitis early in the first few months of life, while some presented with recurrent infections, arthritis, oral ulcers, eczema, folliculitis, and a higher risk of developing B-cell lymphoma, gout, and immune thrombocytopenic purpura [9,10]. Perianal diseases have been reported, some with a severe form of fistulas and abscesses that are comparable to our patient [11]. Our patient presented with typical features of IBD, along with multiple perianal fistulas, fissures, abscesses, diarrhea, fever, and failure to thrive.

Patients with *RIPK1 *mutation noted to have a refractory course poorly respond to conventional treatment that includes immunosuppressive treatments (such as corticosteroids, mesalazine, and azathioprine) and biological treatment (TNF-alpha inhibitor, adalimumab) or surgical management such as colectomy [7,8]. Further, hematopoietic stem cell transplantation was not effective, although it is beneficial in specific genetic defects.

Our patient showed a poor response to medical treatment. Despite undergoing a double-barrel protective ileostomy, his condition did not improve and deteriorated. Perianal disease worsened and became severe with multiple fistulas. Early diagnosis of the mutation could lead to the early start of conventional treatment and prevent any unnecessary surgical procedures. It is important in these scenarios to take the consultant’s opinion for balancing the choice between continuing medical treatment versus an early surgical approach.

## Conclusions

*RIPK1* plays a central role in intestinal homeostasis and regulating inflammasome function. So, RIPK1 protein deficiency due to monogenic mutations should be suspected in patients with VEO-IBD with inflammatory fistulizing features and immunodeficiency. Early diagnosis helps in starting medical treatment which can lead to better outcomes and prevent or delay the need for surgical procedures.
